# Adapting to a US Medical Curriculum in Malaysia: A Qualitative Study on Cultural Dissonance in International Education

**DOI:** 10.7759/cureus.739

**Published:** 2016-08-16

**Authors:** Nicole Shilkofski, Ryan Y Shields

**Affiliations:** 1 Anesthesiology and Critical Care Medicine, Johns Hopkins School of Medicine; 2 Obstetrics and Gynecology, Yale University School of Medicine

**Keywords:** learning styles, international education, cultural dissonance, medical student education, curriculum development

## Abstract

**Context:**

Minimal research has examined the recent exportation of medical curricula to international settings. Johns Hopkins University School of Medicine in Baltimore, USA partnered with Perdana University Graduate School of Medicine in Kuala Lumpur, Malaysia and implemented the same curriculum currently used at Johns Hopkins University to teach medical students at Perdana University. This study aimed to explore the perspectives of first-year medical students at Perdana University, focusing on issues of cultural dissonance during adaptation to a US curriculum.

**Methods:**

In-depth semi-structured interviews with the inaugural class of first-year students (n=24) were conducted, audio-recorded, and transcribed. Two reviewers independently coded and analyzed the qualitative data for major themes.

**Results:**

The most prominent themes identified were the transition from a “passive” to an “active” learning environment and the friendliness and openness of the professors. Students noted that “[Perdana University] is a whole new, different culture and now we are adapting to the culture.” Being vocal during classes and taking exams based on conceptual understanding and knowledge application/integration proved to be more challenging for students than having classes taught entirely in English or the amount of material covered.

**Discussion:**

This study reinforced many cultural education theories as it revealed the major issues of Malaysian graduate students adapting to a US-style medical curriculum. Despite coming from a collectivistic, Confucian-based cultural learning background, the Malaysian students at Perdana University adopted and adapted to, and subsequently supported, the US learning expectations.

## Introduction

Since 2002, a number of US medical schools have collaborated with international institutions to open new medical schools abroad. The goal of many of these collaborations is to contextualize high quality curricula for local epidemiology, while retaining intelligent and talented students in their home countries for training. Over time, these collaborations have the potential to decrease the incidence of "medical brain drain" that is often seen when medical students pursue graduate education in the US, leaving behind medical care systems that are understaffed and stressed from the high burden of disease [1]. These international collaborations provide a unique context to examine the training of future physicians and the impact that culture has on educational processes.

Johns Hopkins University School of Medicine (JHUSOM) in Baltimore, USA partnered with Perdana University Graduate School of Medicine (PUGSOM) in Kuala Lumpur, Malaysia, welcoming the inaugural class in September 2011. PUGSOM represented the first US-style graduate entry medicine program within the country of Malaysia, requiring a Bachelor of Science degree as a prerequisite for admission. All other medical schools in the country (and most others in the Southeast Asia region) predominantly utilize the British model of medical education, in which students enter a five-year medical program immediately after the completion of secondary school. These medical schools offer traditional coursework that has a strongly didactic lecture-based focus, with some curricula in the country also incorporating problem-based learning methodologies and seminars [2-4]. In these programs, teaching is spread over five years with an emphasis on basic medical sciences in the first two years and a gradual transition to clinical skills and hospital-based postings occuring from the third year onward. These schools have curricula that also incorporate courses in language, religion, and other topics that would typically be taught as part of undergraduate degree programs in US universities prior to medical school.

PUGSOM implemented the same Genes to Society (GTS) curriculum used at JHUSOM in Baltimore USA; furthermore, most professors at PUGSOM were either former or concurrent professors at JHUSOM [5]. The GTS curriculum utilizes a developmental organizational structure, is organ system-based, and was developed using the tenets of personalized medicine [6]. The examinations and assessment modalities used at PUGSOM were taken directly from the JHUSOM curriculum with minor changes and contextualization for Malaysian healthcare systems and local epidemiology. Within the GTS curricular paradigm there is a strong emphasis on active learning strategies including peer-to-peer teaching, student-led workshops, team-based learning sessions, and journal clubs. Use of the same curriculum and many of the same professors in this international setting at PUGSOM provided an opportunity to examine how students from a differing educational background adapt to a medical curriculum. 

With the intense demands of medical education and the cultural norms purported through the “hidden curriculum,” medical schools are prime settings to understand how students from varying cultural and learning backgrounds adapt to the environment [7]. There has been ample research regarding the experience of minorities and international students in medical schools around the world [8-10]. Many of these studies demonstrated myriad factors—including language, learning styles, and financial support—contributing to students’ adaptation to a novel educational culture [10].^ ^These factors may all contribute to “cultural dissonance” (an experienced change in the learning environment—impacted by educational formats, classroom design, and other cultural values—which leads to a sense of discomfort and discord). 

While there have been a number of articles about the experience of medical faculty teaching in US medical schools abroad, there is minimal emerging research into the experience of students in these curricula to date [11-13].^ ^This study set out to explore the perspectives of the inaugural class of first-year medical students at PUGSOM, focusing on issues of cultural dissonance in their relationships with professors and adaptation to the GTS curriculum in the multicultural, multilingual society of Malaysia.

## Materials and methods

### Participants

The inaugural class of first-year medical students at Perdana University Graduate School of Medicine participated in the study (N= 24). The students were recruited by email. The class included students of Malay, Chinese, and Indian descent, reflective of the three major ethnic groups that comprise the majority of the Malaysian population, but all students in the study were born and raised in Malaysia. The ages of the students ranged from 23 to 32 years. The students reported universally that English was their second or third language. They came from linguistic, familial, and educational backgrounds where Bahasa Melayu (Malay), Tamil, Mandarin, or Hokkien was their first language.

### Procedure

The study was approved by the Institutional Review Boards of both the Johns Hopkins University School of Medicine and Perdana University Graduate School of Medicine. The qualitative study represented an ethnographic approach to understanding student experience. Each student participated in a one-on-one, in-depth, semi-structured interview that occurred near the completion of the first year of medical school classes during July and August 2012. The investigator who conducted all interviews was a first-year medical student at JHUSOM. This design of peer-to-peer interviews was an attempt to avoid the confounding factor of talking to faculty who were involved in student assessment and evaluation, thus encouraging greater honesty and openness with a fellow student who had experienced the same parallel curriculum at JHUSOM. The interviews lasted between twenty minutes and two hours and were audio recorded in all cases. The interviews were semi-structured and used an interview guide with the same series of questions for all students. The questions were generated through a modified Delphi process based on a consensus panel of professors and faculty members at both PUGSOM and JHUSOM.

### Data analysis

All interviews were subsequently transcribed from audio recordings, de-identified, and coded by two reviewers. The data analysis was approached by a process of open coding followed by axial and selective coding resulting in subsequent theory development. Given the nature of the phenomena under study, i.e. culture and its impact on the learning environment, this type of coding process was necessary to understand the conditions, context, and educational strategies. The data analysis was performed with constant comparative method and coding of the transcriptions of the audio recordings. This was done independently by the two different reviewers who subsequently compared coding categories. The original audiotapes of the interviews remained available for review if needed. The researchers developed categories to group the data early in the process and compared notes and classifications from both individuals. The general coding categories that were used early in the study were setting/context codes, subjects’ perspectives, subjects’ ways of thinking about people and situations, process codes, event codes, strategy codes, and relationship and social structure codes. These open coding categories were then interconnected in the process of axial coding. As observations proceeded throughout the study, selective coding allowed the categories and interrelationships to combine to form story lines about the phenomena under study.

The reviewers used TAMS Analyzer (Text Analysis Markup System, an open source qualitative research tool) to elicit major thematic categories and organize data for each code. The major themes were then discussed between researchers and quotations were drawn from the original data in the manner of “thick description.” In thick description, the voices of interacting individuals are heard from the interviews in order to capture the thoughts, emotions, and web of social interaction among the participants in their operating context.

In keeping with qualitative methodology, triangulation was used to increase validity and search for convergence amongst different sources (i.e. various students) and multiple investigators in order to form themes and categories. During the period of study, the primary investigator spent two months with the students both in and outside the classroom and made informal observations as another form of triangulation, in keeping with an ethnographic approach. These informal observations corroborated many of the conclusions drawn from the data extracted through the in-depth interviews.

## Results

All first-year medical students at PUGSOM (n=24) were interviewed. Twenty-three of the twenty-four students in the class were included in the study and subsequent data analysis. The one international student in the class was excluded from the data analysis because he was not raised or educated in Malaysia and the study focus on cultural impact on education mandated this. Two other students who had attended undergraduate institutions in the US were included in the study as they had extensive experiences within the Malaysian educational system through secondary school.

The major themes developed from the coding process focused primarily on the US style of learning encouraged within the curriculum and the differing relationships with professors in the Malaysian and US systems. The major thematic categories are highlighted and expounded upon below and in Table 1.

**Table 1:** Major themes comparing perceptions about US- and Malaysian-style medical programs Data drawn from in-depth interviews with first-year medical students at Perdana University Graduate School of Medicine

	US Style	Malaysian Style
Classroom experience	Interactive, encouraged to ask questions	Passive listening, discouraged from asking questions
Content focus	Understanding of concepts and integration of knowledge, multiple choice question examinations focusing on knowledge application	Rote memorization of details and minutiae, examinations made up of multiple essay questions focusing on information regurgitation
Relationship with professors	Friends/ colleagues, approachable and open, concerned with all aspects of students’ lives	Hierarchical, no relationship outside of the classroom

### Adapting to an active and interactive classroom

Almost unanimously the students commented on the difference between a “passive” and an “active” learning environment. At the beginning of the year, the students noted, “We started off with our own culture, where we just sit back, relax and enjoy lecture *[sic]*.” This tendency to “just listen and absorb whatever they taught us” stemmed from secondary school and continued with lectures during undergraduate degree instruction in college. One student noted, “We tend to be very passive so [the professors] will talk in front of the classroom and we just listen and we seldom ask questions.”

However, once arriving at PUGSOM, the students felt that the professors would “encourage [them] to ask questions” and “[encourage] more participation.” The professors at PUGSOM “generally want you to speak up and share your opinion,” but in Malaysia “if you’re in class and you’re speaking your mind and telling them ‘hey, I don’t think this is right’ they will think that you’re problematic and that you are rebellious *[sic]*.”

A handful of students worried that adapting to the US ideal of an interactive classroom would prove troublesome when beginning work in Malaysian healthcare systems. They said, “Sometimes we get very subtle reminders about how the Malaysian system is different. Like, ‘you guys are encouraged to question, that [won’t] be welcomed when you go to hospitals … with Malaysian doctors, and if you start to question too much, that’s not going to be easy on you.’”

### US curriculum focus on knowledge integration over rote memorization 

A majority of students brought up the idea that the US curriculum’s focus is not on "memorization or the facts” but more on “understanding the concepts.” The Malaysian undergraduate education system focused on “details” and “if you can regurgitate what [the professors] told you, then you’re a good student.” If you “memorize everything, then you can just do the questions on the exam [and] you can score well.” Yet, “in this American system, you really need to get the bigger picture, not the minor details.”

This difference was also reflected in summative examinations. Many students commented that the Malaysian system exams were “like swallowing all the information and vomiting it out *[sic]*.” Essay questions promoted this method of learning (simply regurgitating what the professor had said during the lecture) while the multiple-choice examinations at PUGSOM required “thinking skills” (critical thinking and application of knowledge to new situations). At PUGSOM the students noticed a difference between questions written by US and Malaysian professors. “The US professors mostly emphasize on the principle, on the big picture. The local [professors], mostly they are very meticulous. Even the questions they ask about [are] rare stuff, rare diseases *[sic]*.”

### Openness and approachability of US professors

The students noted a significant difference between their relationships with professors in the Malaysian and US systems; the US professors appeared much more approachable and friendly than the Malaysian professors. “We have a barrier between ourselves and the professors in the Malaysian culture. So we don’t normally talk to the professors outside classrooms. Here I find that you can connect more, they share things with us.” Many students expressed surprise about and support for the “openness” and “approachability” of professors at PUGSOM. One student reported, “I think the most surprising is that [the US professors are] more close to students—in terms of Malaysian style we don’t get too close to lecturers, because we don’t have this type of environment.”

Another major theme stemming from questions regarding the students’ ­­relationships with professors at PUGSOM was the supportive and encouraging environment. “In the Malaysian culture they like to criticize you rather than compliment you.” But at PUGSOM, “I can feel that they are so concerned about us and what we feel and they really respect our opinion. I think US professors are more encouraging.”

However, a few students expressed fears of feeling over-supported without adequate constructive criticism. “I’m just worried that people say we’re being pampered. Because … [previously] we had to learn to adapt to the teaching style of the professor, not the other way around. Now the professors [here are] adapting to our learning style.” A number of students felt that the professors “keep telling us the nice things, but [we want to know] what are the bad things too *[sic]*.” 

### Lack of hierarchical structure in a traditionally hierarchical culture

In addition to the approachability of the professors at PUGSOM during lectures, students noted that “the relationship is more like friends or colleagues, rather than teacher and student relationship *[sic],*” even outside the classroom. In the Malaysian system, “for 20, 22 years … we have this relationship with teachers that is very distant. We never really talk about problems with teachers or lecturers. And normally in our culture we do not question our lecturers. They are always right. As a student, whatever the lecturer says you just accept it. So you do not question them, do not stop them when they are lecturing. That is the style of teaching we are used to. Even from primary school, secondary school. So it’s very different [at PUGSOM], very surprising actually *[sic]*.”

Once arriving at PUGSOM, the students felt that “with the American faculty … we do not know how to deal with them— we’ve never been taught by them. So we follow their lead and the way they did it with us was call us by our first name, you are our friend, you are our equal, and we are here to teach you as future colleagues, that’s how they treat us. So they set the trend in that way and we are following suit.” The students noted a sense of “culture shock” coming to PUGSOM, adapting to “calling professors by their first name” and professors being “friendly-like peers.” 

The students were also surprised by the interest and concern professors had in their lives outside of school. The students expressed that the professors recognized that “we do not only study, we have family issues, we have friends, and we have our social life. So if there is any problem, we can always go to any one of them.” The students sought advice from professors for issues related to both school and life. One student who struggled during the first semester noted that a professor’s guidance was “not only tutoring in terms of anatomy, how to answer questions correctly, but also other sorts of advice—that it’s ok because downfalls like this happen throughout your life and you can’t just step out of it, you have to go through it *[sic]*.”

## Discussion

Individual learning styles and cultures of learning have been extensively covered in both the education and medical education literature [14-17]. Joy and Kolb explicitly examined how culture influences individual learning styles, focusing on “abstract conceptualization” versus “concrete experience” learning styles in high- and low-collectivism cultures [16]. These theories were further borne out in studies and interviews comparing Eastern cultures (i.e. Chinese and Korean with strongly Confucian-based academic backgrounds) and Western students in various classroom environments [18-21]. In medical education, studies have shown vastly different approaches in the learning of anatomy between these two groups [22-23].

Recognizing that learning exists in a cultural and social context aids both educators and learners in negotiating classrooms and possible new approaches in education. However, learning styles and cultures are never static, simple, or without internal diversity [14]. In fact, studies have shown that the area of specialization can have a larger impact on learning style than the broader cultural context [16]. This becomes especially significant in the field of medicine, with its own collective culture, language, and hidden and implicit curriculum. While a recent study from a medical school in the Middle East using a “transplanted curriculum” has shown a dominance of individual agency as a determinant of learning style, it is critical to examine these cases within the specific cultural context [13]. 

This study reinforced many of the cultural education theories of learning styles by revealing the major issues of Malaysian graduate entry medical students adapting to the first year of a US-system medical curriculum [16, 24]. Though the Malaysian students at PUGSOM came from a collectivistic, Confucian-based cultural learning background, they adopted and adapted to US learning expectations and values including a more Western orientation towards individualistic learning, being vocal in the classroom, and self-expression. 

The transition into an education system that emphasized an understanding of concepts over the rote memorization of details reinforced the students’ need to ask questions. A study of Bahraini students and their intercultural transition to medical school highlights the concern of teachers that students use memorization of theoretical information as a primary strategy in their secondary education programs, which can lead to “spoon-feeding pedagogies” [13]. As seen previously in the literature, an interactive classroom further encourages students to respectfully question professors and gain confidence in verbalizing and voicing their confusions and concerns, challenging the students’ past cultures of learning [24-25]. This paradigm shift from a teacher-centric approach seen in traditional school practices to a learner-centric approach has been the subject of prior studies in students’ cultural transition to medical school [10, 13]. In our study, this shift in academic approach created a sense of cultural cognitive dissonance for many students and a number of students, without prompting, described the transition as “a culture shock.” Adapting to asking questions in class and taking exams based on conceptual understanding and knowledge integration proved to be the most challenging aspects of their first year, more so than having classes taught entirely in English or the amount of material necessary to study.

The approachability of the US professors led to a system where the students and professors were more “collegial” as opposed to the typical hierarchy found in the Malaysian and other Asian education systems described in the literature [26-27]. The support and care provided by the professors at PUGSOM surprised the students and allowed them to gain a more profound relationship with the professors. It should be noted, however, that we cannot conclude this perceived collegial relationship was due to the curricular structure or pedagogies used by the professors. It is likely that the small class size and unique nature of PUGSOM contributed to fostering the perceived lack of hierarchy and impacted the interactions between the students and the professors.

The two major differences between the Malaysian and US system curricula reinforced one another. The openness of the professors helped to create an environment where asking questions was not only encouraged, but expected. The close relationship with the professors at PUGSOM facilitated a context where the students felt sufficiently at ease to speak up in class, seek out academic help, and ask for advice on personal issues not related to medical school, thus fostering a true learning community. This apprenticeship and mentorship by faculty members within the medical school community helped to foster a socio-cultural “Community of Practice or CoP,” which was previously used in an educational context by Hayes, et al. as a lens to understand intercultural transition [13]. These findings are also supported by prior research that demonstrated high learning environment (LE) ratings at PUGSOM that was driven by student responses on various LE scales within domains that included "perception of teaching," "meaningful engagement," and "acceptance and safety" [28].

### Limitations

One of the inherent limitations of this study is that the study population was comprised of only one class of medical students at one university. However, all students in the class were interviewed and the major themes elicited reached a point of saturation. In spite of being interviewed by a fellow medical student and explicit reassurances of anonymity, the students may have felt restrained by perceived ties of the interviewer to the university. External validity is restricted by country and cultural contexts, specific medical curriculum, and limited sample size. In addition, as mentioned above, some of the results and student comments could potentially be impacted by or reflective of the small class size of the student body, the type of faculty who self-selected to participate in the educational process, and the novelty of the graduate-entry model of education within Malaysia, rather than of the curriculum itself.

## Conclusions

This study, which focused on only one example of a US medical curriculum being used abroad, helped to illustrate the perspectives of students adapting to a novel educational system and provided an in-depth look at how US medical curricula can be exported and adapted for use abroad. It also took into account cultural competency and student learning styles as factors in the acculturation of students into the field of medicine. Almost all the students in the study advocated the use of a more interactive classroom style and the presence of supportive and approachable professors, though the students came from a very different learning culture and pedagogy. Given the perceived advantages of the novel learning environment, students voiced willingness to adapt to a culturally dissonant style.​ With this endorsement, a US curricular model can be tried and evaluated through further exportation of medical curricula abroad. As the students become physician educators themselves, one may see an evolving teaching style disseminated throughout the country in a sustainable manner. Further research with subsequent classes of medical students at other medical institutions abroad using US curricula would aid in reinforcing the use of a US curriculum to educate physicians in vastly different cultural and educational contexts. Moreover, it would be valuable to follow changes in personal learning styles of students over four years of medical school education and observe how US professors subsequently modify their own teaching styles. Additional qualitative research into the experiences of PUGSOM students as they integrate into the Malaysian healthcare system as postgraduate trainees would also provide helpful insight into navigating cultural dissonance longitudinally throughout the course of medical education. Ultimately, evaluating how PUGSOM students approach medicine and patients within a Malaysian context would demonstrate the inherent benefits or risks of culturally different pedagogies.
